# Ballistic Impacts with Bullet Splash—Load History Estimation for .308 Bullets vs. Hard Steel Targets

**DOI:** 10.3390/ma16113990

**Published:** 2023-05-26

**Authors:** Riccardo Andreotti, Andrea Casaroli, Ivan Colamartino, Mauro Quercia, Marco Virginio Boniardi, Filippo Berto

**Affiliations:** 1 Department of Mechanical Engineering, Politecnico di Milano, Via La Masa 1, 20156 Milano, Italy; andrea.casaroli@polimi.it (A.C.); ivan.colamartino@polimi.it (I.C.); marco.boniardi@polimi.it (M.V.B.); 2 Callens® AREA3, Via Merini 37, 21100 Varese, Italy; mauro.quercia@area3.it; 3 Department of Chemical Engineering, Materials and Environment, La Sapienza University of Rome, Via Eudossiana 18, 00184 Roma, Italy; filippo.berto@uniroma1.it

**Keywords:** bullet splash, bullet-splash, ballistic impact, terminal ballistics, finite element method, ballistic protection, explicit solver, impact simulation, Creusabro^®^, Durostat^®^

## Abstract

The study focuses on testing a simplified way of estimating the resultant force due to ballistic impacts resulting in a full fragmentation of the impactor with no penetration of the target. The method is intended to be useful for the parsimonious structural assessment of military aircrafts with integrated ballistic protection systems by means of large scale explicit finite element simulations. The research investigates the effectiveness of the method in allowing the prediction of the fields of plastic deformation collected by hard steel plates impacted by a wide range of semi-jacketed, monolithic, and full metal jacket .308 Winchester rifle bullets. The outcomes show the effectiveness of the method being strictly related to the full compliance of the considered cases with the bullet-splash hypotheses. The study therefore suggests the application of the load history approach only after careful experimental investigations on the specific impactor–target interactions.

## 1. Introduction

For two decades, transient finite element simulation has represented a fundamental tool for the crashworthiness assessment and structural optimization of vehicles [1,2,3]. In the aerospace industry, airframe development passes through many steps of optimization against potential threats for the survivability of the vehicle such as bird impacts, hail impacts, emergency landing, ditching, and wire strike. All of these potential threats are faced by means of dedicated simulations involving extended parts or even the entire airframe, modelled in finite elements to verify the survivability of every layer of material and every single rivet connecting the structures. In this technological scenario, the development of a modern military aircraft follows the same methodological approach with the obvious complications due to the main aim of a military vehicle: to maximize the probability of surviving a real combat scenario. This implies that the critical components of the vehicle must be protected against the typical threat of warfare: ballistic impacts [4,5]. To do this, critical areas of the vehicle, such as the cockpit and the transmission, are surrounded by ballistic protection panels, which are experimentally verified to be able to withstand specific ballistic impacts without allowing any penetration of the bullets. In this field the aim of the simulations is therefore not to verify the ability of the protective panels to withstand the impact of the bullets but instead to verify the strength of the support structures to which the panels are attached against the impulsive loads due to several impact positions along the entire airframe, representing the random incidence of the shots during fire fights. In this scenario it is therefore fundamental to be able to model the stress propagation through the structure to verify the strength of every connection and part of the support system. This involves many different load cases involving significant parts of the airframe modelled in full detail.

Protective panels typically consist of an external layer of hard ceramic tiles intended to fragment the hitting bullets during the first phase of the impact, and an internal layer made of composite fabric to withstand the local impact forces and absorb the residual energy without allowing the bullet fragments to penetrate the protection panel [6]. Therefore, the typical scenario of a perfectly working protection panel consists of a total fragmentation of the bullet with no penetration of the target. This phenomenon is called bullet splash, due to the fact that the fragmentation of the bullet makes its kinematics similar to the flow of a mass of a fluid being deflected by the target surface [7,8]. In this scenario Andreotti et al. (2021) first proposed a simplified approach to reduce the computational cost needed to simulate the forces acting on the target due to bullet splash, by considering the phenomenon as a fluid-structure interaction where a mass of fluid represents the behavior of the bullet fragments. The approach proved to be effective in predicting the local and global effects of bullet splashes due to 9 × 21 mm full metal jacket bullets hitting 4 mm AISI 304 plates [9]. Based on that experience, Andreotti et al. (2022) [10] proposed and validated a further simplified method to make the finite elements simulations more efficient and equally effective by avoiding the hydrodynamic part of the calculation, by introducing an estimated load history approach that consists in decoupling the bullet fragmentation phenomenon and the target reaction to avoid the costs of modelling the bullet, and introducing an equivalent impact force as a load curve *F(t)* to be directly applied to the impact point on the structure; the intensity vs. time curve is calculated based on the initial impact velocity and the density distribution of the specific bullets. The approach proved to be effective and extremely efficient for 9 × 21 mm full metal jacket bullets hitting 4 mm AISI 304 plates as well as for monolithic copper .308 rifle bullets hitting high hardness steel plates. The validation confirmed the equivalence between the fluid structure interaction and the load history approach in terms of stress waves propagating from the epicenter of the target, resultant forces at the constraint, and residual deformation fields of the plates [11,12].

In this paper we propose the application and experimental validation of the estimated load history approach to cover a wide range of rifle bullet typologies, from monolithic to partially jacketed and full metal jacket bullets. The validation has been performed by comparing the residual deformation field measured on steel plates impacted by the bullets and the residual deformation field predicted by finite element simulations where the applied load history was estimated based on experimental measurements of the impact velocity and density distribution of the tested bullets. The work gives the reader a reference for the effective applicability of the load history method for finite element simulations intended to simulate the stress propagation from ballistic protection panels to the support structures to assess, without the need to model the bullets, their fragmentation, and the interactions with the targets, therefore allowing a more parsimonious approach to the problem. This method is based on the hypothesis that the protective panel is able to withstand the ballistic impacts of the considered bullets at a certain velocity, resulting in a complete fragmentation of the bullet with no penetration or fragmentation of the target. Within these hypotheses the method consists in estimating the force history due to the impacts based only on the bullet sections and their impact velocity, and applying the estimated load history directly to the elements of the structure to simulate the structural phenomenon. Section 2 provides the reader with all the information to reproduce the conducted tests. In Section 3 we provide all the main analytical, numerical, and experimental results involved in the validation process. In Section 4 the results are discussed. In Section 5 we summarize the conclusions and further developments of the research.

## 2. Materials and Methods

To validate the load-history approach on a wide range of bullet typologies, we took into consideration four different .308 rifle bullets hitting dual-layered steel plates made of two different steels. Both steels have high strength and significant ductility so that they can withstand the impacts without allowing the penetration of the bullets, and, at the same time, are able to collect residual deformations so that the field of residual displacements could be used as experimental evidence to compare with the numerical results for the validation of the approach.

### 2.1. Ballistic Test Procedures

**Figure 1:**
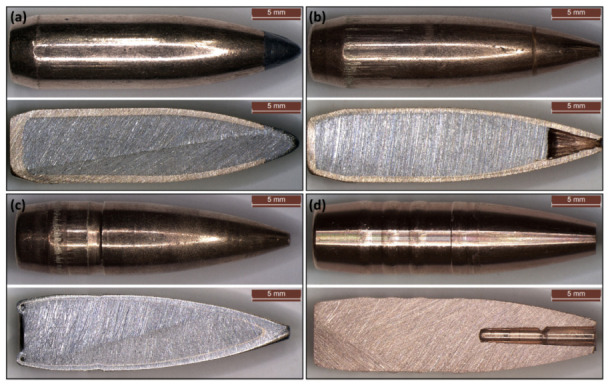
External geometry and longitudinal section of the bullets used for experimentation; (**a**) Soft Point, (**b**) Hollow Point, (**c**) Full Metal Jacket and (**d**) Monolithic.

The bullets were shot using a Remington 700 cal. 0.308 Winchester. The initial bullet velocity was measured using a Magnetospeed^®^ V3 ballistic chronograph assembled at the rifle muzzle. Four different caliber .308 Winchester types of bullet were used: Soft Point (SP), Hollow Point (HP), Full Metal Jacket (FMJ) and Monolithic (MONO) bullets. Their main characteristics are summarised in Table 1. The external geometry and longitudinal section of the bullets was documented through a LEICA^®^ M165 stereo microscope at 3.5× magnification. The bullets were sectioned using 120 grit abrasive paper, lubricated using water (Figure 1).

The targets were obtained by joining two 4 × 500 × 500 mm plates. Two different high-strength structural steels were tested; their mechanical and metallurgical properties are detailed in Section 2.2. Four tests were carried out on each metal plate using the bullets detailed in Figure 1 and in Table 1. The nominal impact positions are detailed in Section 2.3.

The targets were positioned at 100 m from the rifle muzzle, oriented normally to the bullet trajectory. The metal plates were bolted to a steel and wood support frame specially fabricated to carry out the tests. The support frame was designed to guarantee full visibility of the metal plate side from the cameras used to film the impacts. A panel with a chessboard pattern composed of black and white squares with a 20 mm side was installed on the opposite side. This solution was adopted to guarantee a dimensional reference for the high frequency camera shootings.

Upon completion of the tests, the bullet impact areas were accurately photographed both on the front and rear sides of the metal plates; the pictures are provided in Section 3.1. The dome-shaped bulges created by the bullets in the rear surface of the metal plates were also measured using a Borletti dial gauge with a sensitivity of 10 μm. The measurement procedure included tracing four reference lines slanted by 45° relative to each other. All the lines cross the apex of the bulges which was adopted as zero point. 14 measures of metal plate deformation were taken for each of the four lines, at a distance of ±5 mm, ±10 mm, ±15 mm, ±25 mm, ±35 mm, ±45 mm, and ±55 mm from the bulge apex. The values so obtained were plotted in diagrams (Section 3.4), each showing the minimum and maximum deformation of the metal plate measured at increasing distance from the bulge apex.

### 2.2. Mechanical and Metallurgical Characterisation of the Metal Plates

The targets were fabricated using high-yield stress structural steel plates. The two steel grades selected for the experimentation go under the trade names of Creusabro^®^ 8000 [13,14,15] and Durostat^®^ 400 [16]. They are characterised by a combination of very high tensile strength and optimum wear resistance. Their typical applications are ballistic shields and barriers, buckets for heavy equipment, dump truck beds, conveyor components, and rollers and rotors for grain mills and oil mills. The high level of hardness and tensile strength is obtained through controlled cooling after hot rolling. Their limited carbon content, together with a considerable manganese content (or manganese-nickel content in the case of Creusabro^®^ 8000), allows them to have acceptable toughness and weldability characteristics, notwithstanding very high values of hardness and tensile strength. The significant amount of chromium and molybdenum guarantees the hardenability necessary to form a mixed martensite and bainite structure. Both steel grades are characterised by micrometric carbides finely dispersed in the metal and further improving abrasive wear resistance [14].

In comparison with Durostat^®^ 400 steel, which is composed exclusively of martensite, bainite and carbides, Creusabro^®^ 8000 steel also exhibits a small amount of residual austenite that turns into martensite when it is subjected to cold deformation. This property, known under the acronym TRIP (Transformation Induced by Plasticity), further improves the wear resistance and impact strength of Creusabro^®^ 8000 steel compared to Durostat^®^ 400 steel. The TRIP effect enhances steel hardness and its ability to absorb energy as the cold deformation increases [17,18].

The chemical composition of both steels was assessed by means of optical emission spectroscopy (OES). Table 2 and Table 3 provide an overview of the results. Both steels meet the requirements of the applicable steel mill specifications. Comparing the two steels, it is clearly apparent that Creusabro^®^ 8000 is much richer in alloying elements than Durostat^®^ 400. Durostat^®^ 400 does not contain molybdenum, and its carbon, chromium, and silicon content is less than half of the values measured in Creusabro^®^ 8000. On the other hand, Durostat^®^ 400 contains twice the amount of manganese, but it does not contain nickel which is present in a good quantity in Creusabro^®^ 8000. The chemical composition clearly shows that Creusabro^®^ 8000 is by far more hardenable but less weldable than Durostat^®^ 400, compared to which it exhibits an equivalent carbon content that is higher by approximately 25% (CECreusabro^®^8000 = 0.63; CEDurostat^®^400 = 0.50). Having received the same heat treatment, Creusabro^®^ 8000 is also harder and mechanically stronger thanks to its carbon content that is more than twice that of Durostat^®^ 400.

Both metal plates were subjected to metallographic analyses and Vickers HV1 hardness tests. The samples were mirror polished using progressively finer abrasive paper (120 grit, 180 grit, 320 grit, 400 grit, 600 grit, and 1200 grit) and polishing cloths with diamond-based synthetic abrasive (grain size 3 µm and 1 µm). Lubrication was guaranteed using water with abrasive paper and the suspension containing the abrasive matter with the cloths. Metallographic etching was obtained using the reagent “Nital 2”, a 2% nitric acid/ethanol solution. The pictures were captured using a LEICA^®^ DM4000M optical microscope at 500× magnification. The same samples were also used to evaluate core hardness in the metal plates. The tests were conducted using a Vickers LEITZ^®^-WETZLAR^®^ (Leica Camera AG, Wetzlar, Germany) micro-durometer fitted with a digital camera and set at the HV1 hardness scale (1 kgf, 30 s).

**Figure 2:**
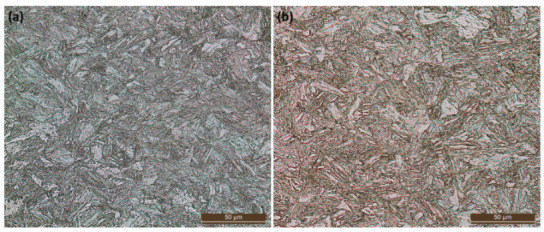
Metallographic images of the metal plates used for experimentation, made of (**a**) Creusabro^®^ 8000 steel and (**b**) Durostat^®^ 400 steel. Both steel grades form martensite and bainite with carbides finely dispersed in the metal (etching agent: Nital2—Magnification: 500×). This type of investigation could not reveal the small amount of residual austenite prescribed for Creusabro^®^ 8000 steel.

The tests confirm what was previously suggested by the chemical composition, i.e., both steel grades form martensite and bainite with carbides finely dispersed in the metal (Figure 2) [19]. This type of investigation could not reveal the small amount of residual austenite prescribed for Creusabro^®^ 8000 steel that, in any case, exhibits an average hardness higher than Durostat^®^ 400 (540 HV1 compared to 420 HV1).

**Figure 3:**
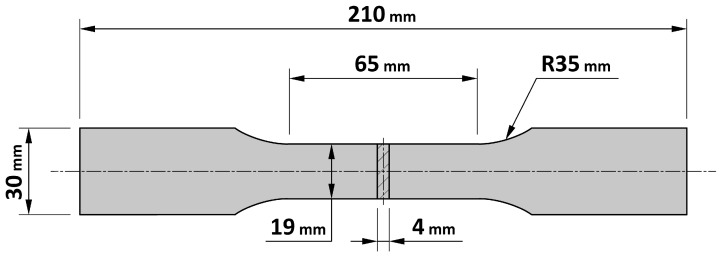
Geometry of the specimens used for the tensile tests. Proportional specimens obtained in compliance with the UNI EN ISO 6892-1:2020 standard were used [20].

**Figure 4:**
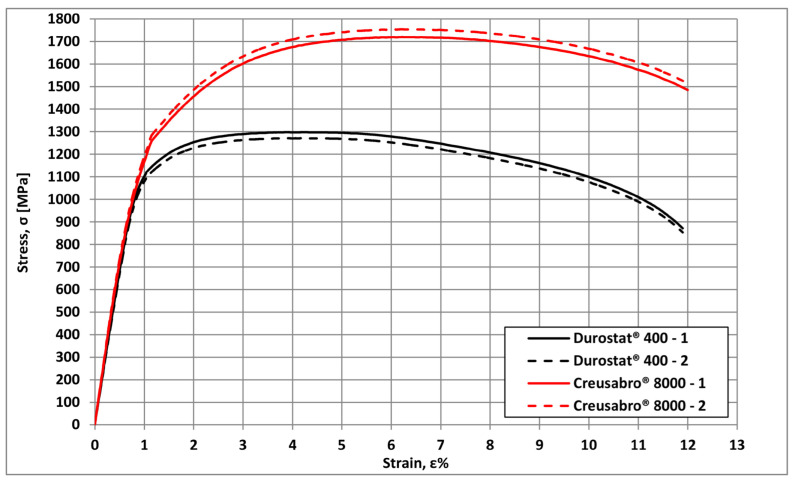
Engineering stress/strain curves for Creusabro^®^ 8000 steel (red lines) and Durostat^®^ 400 steel (black lines) plates used for experimentation.

The characterisation of the two steels was completed by conducting tensile tests at room temperature. Proportional specimens, obtained in compliance with the UNI EN ISO 6892-1:2020 standard, were used [20,21] (Figure 3). Results are summarised in Table 4, while the engineering stress/strain curves are shown in Figure 4.

The tensile tests confirm what had been anticipated by the hardness tests. The ultimate tensile strength and yield strength of Creusabro^®^ 8000 exceed those of Durostat^®^ 400 steel (UTS_Creusabro_^®^_8000_ = 1735 Mpa; YS_Creusabro_^®^_8000_ = 1190 Mpa; UTS_Durostat_^®^_400_ = 1290 Mpa; YS_Durostat_^®^_400_ = 1100 Mpa). The YS/UTS ratio and percentage elongation under maximum load, Ag%, are, respectively, equal to 0.85 and 3% in Durostat^®^ 400 and 0.68 and 5% in Creusabro^®^ 8000. Both steels exhibit analogous values of percentage elongation after fracture (A%_Creusabro_^®^_8000_ = A%_Durostat_^®^_400_ = 11%). These values confirm another important aspect revealed by chemical analyses: Creusabro^®^ 8000 steel’s strain hardening ability is higher than that of Durostat^®^ 400 steel. Creusabro^®^ 8000 is also superior to Durostat^®^ 400 in terms of plastic deformation ability before reaching the necking point. Therefore, the Creusabro^®^ 8000 steel exhibits superior performance compared to the Durostat^®^ 400 steel in terms of tensile strength, hardness, and energy absorption capability. On the other hand, its weldability is poorer than in Durostat^®^ 400.

### 2.3. Testing Setup and Impact Speed Computation

**Figure 5:**
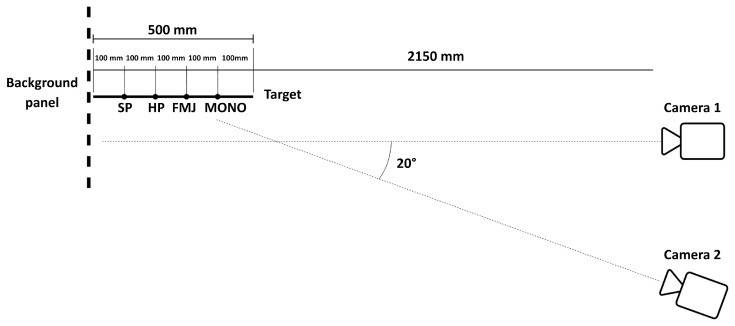
Scheme of the camera settings (top view).

**Figure 6:**
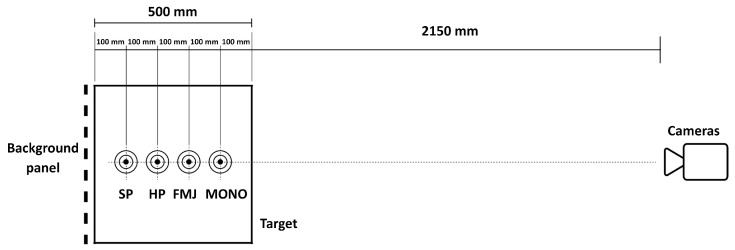
Scheme of the camera settings (front view).

Two Phantom VEO 710 high speed cameras were used to capture the bullets’ speed and trajectory. In particular, the first was positioned orthogonally with respect to the trajectory at a distance variable between 2250 and 2550 mm from the trajectory plane; the second one, instead, was positioned at the same orthogonal distance with an angle of 20° to obtain a perspective view of the shots. Data were acquired via two ethernet cables with a synchronized digital trigger. Further details for each shot are shown in Table 5 and Table 6, while schemes of the data acquisition setup are shown in Figure 5 and Figure 6.

**Figure 7:**
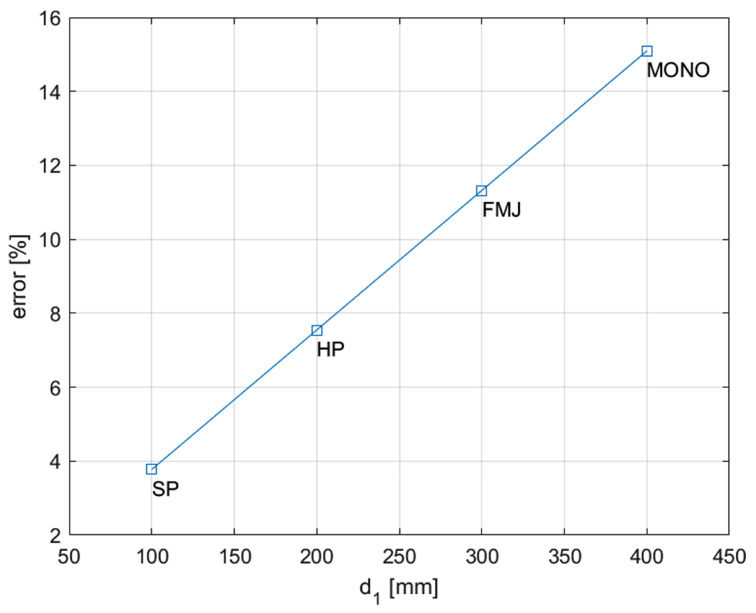
Error on position vs. observer-reference distance. The worst-case scenario is identified for the Monolithic bullets, for which the distance from the trajectory and the reference plane is 400 mm: the apparent speed in such cases is 15.1% higher than the real speed.

While the perspective view was meant to capture the three-dimensional fragmentation dynamics of the projectile, the orthogonal videos were recorded to compute the projectile impact and exit speeds, via the well-known time-of-flight principle: knowing a priori the dimensions of a reference object, the video’s resolution, and time history, the projectile trajectory is easily transformed into impact velocity. Such a method is one of the most used in ballistics to effectively evaluate the trajectory of bullets [22], with multiple applications in 2D and 3D analyses [23]. In the present work, even if the bullet itself could have been in principle used as reference for calibration, its small dimensions would have introduced a potentially high error; consequently, the reference object was a background reference plane, a chessboard panel attached perpendicularly to the frame. As described in Section 2.1, the chessboard was characterized by regular squares of 20 × 20 mm each, while the nominal trajectories were driven by four equally spaced targets in correspondence of the vertical midline of the plates. To minimize the error, the calibration procedure was performed on the highest possible number of squares, resulting in 0.8 mm/px. Once the calibration is performed, the bullet speed is computed by evaluating the distance travelled by the bullet and dividing it by the correspondent time interval. However, the fact that the reference plane used for calibration and the projectile trajectory plane were not coincident introduced a distortion factor between the measured and real speeds. The issue is easily solved if the position of the camera is fixed with respect to the reference plane: in that case the distortion factor is linear as a function of the distance of the bullet from that plane (Figure 7) and consequently the correction is straightforward.

Naming this distance $d_{1}$ and the observer-reference plane distance $d_{2}$, a correction factor *C* can be defined as follows:

(1) $$C=1-\frac{d_{1}}{d_{2}}$$

Consequently:

(2) $$v=Cv_{meas}$$

Considering the relatively low resolution and number of available frames capturing the bullet during the pre-impact phases, multiple measurements were carried out mapping the position of three sections of the bullet (frustum, beginning of the ogive, and tip) for multiple combinations of frames. This redundant procedure allowed us to average out the human error introduced by manual recognition of the projectile shape. A minimum of six measures per shot were collected and averaged to obtain the resulting impact velocities.

### 2.4. Bullet-Splash Load History Estimation

The load history estimation is based on two main hypotheses that must be verified experimentally:

- The bullet encounters complete fragmentation during the interaction with the target;
- The target can withstand the interaction without being penetrated, causing the deflection of the debris.

The load histories were calculated according to the progressive fragmentation theory introduced by Andreotti et al. in 2022 [10,11,12] for 90 degree bullet splashes. The load history formula allows us to reconstruct the *F*(*t*) depending on the density distribution characterizing each section of the bullet and its impact speed. The interaction between the impactor and the target is treated as the interaction of a fluid flow, representing the flow of bullet debris, and a rigid, fixed plane normal to the axis of the bullet. The force at a generic time *t* is calculated as the time derivative of the elementary portion of the bullet ideally intersecting the target surface at that time. Considering the 90-degree deflection, the elementary variation in momentum can be expressed as:

(3) dq=dmv

where *v* is the impact velocity of the bullet and *m* is its mass. Considering a homogeneous bullet with density *ρ*, assuming that the velocity variation only happens to the material ideally intersecting the impact surface, the elementary mass that is deflected in the elementary time *dt* is:

(4) dm=ρA(t)vdt

where *A(t)* represents the intersection between the bullet volume and the impact surface plane, and *vdt = ds* is the elementary translation of the bullet in *dt*. By substituting Equation (4) into Equation (3), and dividing both terms by *dt*, we obtain the expression of the impact force for a homogeneous bullet:

(5) $$F(t)=\frac{dq(t)}{dt}=\rho A(t)v^{2}$$

which integration in time correctly equals the initial momentum of the impactor:

(6) q=∫F(t)dt=vρ∫A(t)vdt=vρ∫A(s)ds=vρV=vm

where *V* is the volume of the homogeneous bullet.

For a generic impactor, composed by *M* materials, the load history due to the bullet splash can therefore be expressed as the sum of *M* terms:

(7) $$F(t)=\sum_{i=1}^{M}F_{i}(t)$$

Therefore, the generic expression of the estimated load history due to bullet splash is:

(8) $$F(t)=v^{2}\sum_{i=1}^{M}\rho_{i}A_{i}(t)$$

where $\rho_{i}$ is the density of the *i*-th material, and *A_i_(t)* is the section of that material ideally intersecting the surface of the target at the interaction time *t*.

To estimate the load-history due to bullet-splash of the tested impactors the formula (Equation (8)) was applied to the bullets section (Figure 1) by imposing an impact speed equal to those experimentally measured for the tested impacts.

To acquire the *A_i_(t)* of the bullets, the first step consisted in mapping its sections normally to the axis of the bullet *x*. At *N* discrete axial coordinates *x_j_* (30 to 50 points along the entire axis of the bullets, depending on their geometrical complexity) the radial coordinates of every material boundary were measured, so that, in general, the area of the i-th material at axial coordinate *x_j_* could be calculated as the area of a hollow circle:

(9) Ai(xj)=π(Reij2−Riij2)

where *Re_ij_* and *Ri_ij_* are the external and internal radiuses of the *i*-th material at the *j*-th axial coordinate *x_j_*.

Once the materials sections *A_i_(x)* were mapped, the *A_i_(t)* of the specific shot was calculated by converting the spatial coordinate into time coordinates, by dividing the *x_j_* by the initial impact velocity *v*:

(10) $$A_{i}(t_{j})=A_{i}(\frac{x_{j}}{v})$$

The resulting load history is therefore a discretized curve to be automatically interpolated by the finite element solver at every time-integration step:

(11) $$F(t_{j})=v^{2}\sum_{i=1}^{m}\rho_{i}A_{i}(t_{j})$$

### 2.5. Finite Element Simulation Setup

**Figure 8:**
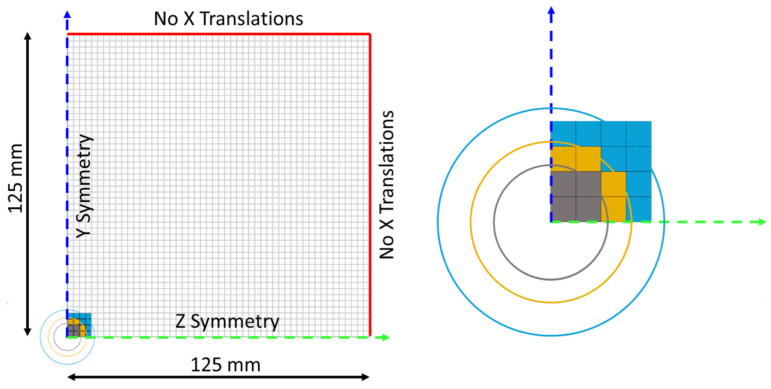
Overview (**left**) and detail (**right**) of the finite element model of the plate highlighting the choice and progressive increase in the loaded elements characterizing the three arbitrary load distributions: 100 mm^2^ distribution (grey), 200 mm^2^ distribution (yellow), and 400 mm^2^ distribution (light blue); the three circles represent the area-equivalent circular extensions corresponding to the minimum and maximum limit of the compression area, and the external limit of the sliding area as experimentally observed on the impacted plates, respectively (Figures 12 and 13). Only a quarter of the plate was modeled, with orthogonal symmetry conditions.

To verify the representativeness of estimated impulses, the considered impacts were simulated by applying the load histories to the plates by means of the explicit finite element solver LS-DYNA [24]. The plates were uniformly discretized with 2.5 mm fully integrated 4-node shell elements with 16 integration points in the thickness. The load was applied normally to the plates. Each shot was simulated by applying the corresponding load history as uniformly distributed on arbitrary areas, centered at the epicenter of the impacts, and with extensions comparable with the extension of the interaction marks visible on the impacted plates. The actual space distribution of the pressure fields acting during the impacts is in fact unknown. Therefore, to verify the sensitivity of the simulation results to the local pressure distribution, the same load history was applied on different arbitrarily defined distribution areas. The shots were simulated three times, by varying the extension of the loaded surface according to the experimental observations (Figures 12 and 13). The different tested areas had 100 mm^2^, 200 mm^2^, and 400 mm^2^ extensions (see Figure 8), representing, respectively, the minimum and maximum extension of the compression area, and the extension of the sliding area as experimentally observed on the impacted plates (Figures 12 and 13). The plates were constrained in the load direction *x* by zero translation of the boundary nodes, and with double orthogonal symmetry planes intersecting at the epicenter of the impacts. The constraint between the two plates was assured by setting a penalty contact with a static and dynamic friction coefficient of 0.65, typical for clean dry steel-on-steel interactions [25].

#### 2.5.1. Development of the Finite Element Model

The development of the finite element model here proposed was the result of three main steps, aiming at finding the most efficient discretization strategy to eventually allow engineers to correctly simulate the propagation of the stress waves caused by bullet splashes on generic, extended, ballistic protection panels. The development started from a highly detailed simulation of the local effects of the impacts, and progressively evolved towards more parsimonious models guaranteeing the same effectiveness in terms of predicting the residual deformation fields and the stress waves generated by the impacts.

The first step (Andreotti et al., 2021 [9]) consisted in simulating the bullet-splash phenomenon as a fluid structure interaction (FSI) to reproduce the progressive deflection of the bullet fragments interacting with the target. The model used for the FSI simulation was a 3D solid mesh composed of 0.3 mm hexahedral elements. The experimental validation of this model was based on the measurements of the micro-hardness and deformation fields across the impact surface of 4 mm AISI 304L steel plates impacted by 9 × 21 mm FMJ bullets. The validation demonstrated extreme accuracy in predicting the field of plastic strain as well as the overall residual deformation field.

The second step introduced the load history estimation method discussed in this paper. The method was first tested on the same experimental dataset analyzed by Andreotti et al. (2021) [9]. The simulation applied the estimated load history as a uniformly distributed load on arbitrary concentric circular areas, with the plate being discretized with the same 0.3 mm hexahedral elements mesh used for the validation of the FSI approach. The comparison between the overall residual deformation field, the stress waves, and the resultant reaction forces validated the load history approach as an equally accurate way to simulate bullet-splash for macroscopic structural assessment purposes, allowing a reduction of the computational cost of 60% compared to FSI.

In the third step, to improve the efficiency of the method and its parsimony and suitability to real-world engineering applications, a progressive simplification of the structural model was introduced, leading to the full shell model here proposed. The validation of the 2.5 mm shell model was performed again on the 4 mm AISI 304L steel plates impacted by 9 × 21 mm FMJ bullets; the residual deformation field, the stress waves, and the resultant reaction forces predicted by the simulation were confirmed to be accurate in comparison with the experimental evidence and with the results of the most accurate simulation based on the 0.3 mm size solid finite element model (Andreotti et al., 2022 [10]).

Compared to the FSI simulation model, the 2.5 mm shell discretization allowed a reduction of the calculation cost of more than 99%, also guaranteeing accurate prediction of the structural effects of the impacts, thanks to a maximum stable integration time step of 0.46 µs, that guarantees an accurate reproduction of the considered impulses, whose duration is less than 50 µs, and is compatible with reasonable computational costs for real-world applications. The analyses discussed in the present paper were conducted with an initial time integration step equal to 90% of the maximum, i.e., 0.414 µs. It is important to notice that the 2.5 mm size was also identified as the maximum element size compatible with reasonable load application detail to distinguish the load distributions considered in the study. The optimization of the computational cost of the finite element model also considered the symmetries and the extension of the discretized plate. Preliminary tests were conducted on the entire model to verify the influence of the boundary effects on the different shot positions, concluding that no boundary effects were significant on the results; therefore, the overall dimensions of the finite element models of the plates were reduced to 125 × 125 mm, with two orthogonal symmetry planes, to represent a 250 × 250 mm plate impacted at the center. In fact, the dimensions of the plates are such that the local effects of the impacts cannot be influenced by the boundary constraints because the maximum duration of the considered load history is less than 50 µs while the time for the stress waves to travel back and forth from the epicenter to the constraints is around 100 µs (considering the sound speed in steel is equal to 5000 m/s).

#### 2.5.2. Constitutive Model Associated with the Plates

The constitutive model associated with the plates is a kinematic elastic-plastic model with damage (*MAT_81\*MAT_PLASTICITY_WITH_DAMAGE in Ls-Dyna kewords [26]). For Creusabro^®^ 8000 the post-yield hardening is regulated by a constant modulus ETAN = 11,891.7 MPa; the initial necking strain is EPPF = 0.05827. For Durostat^®^ 400 the hardening modulus is ETAN = 5759.59 MPa; the initial necking strain is 0.04402. The rate effect was implemented as a scale factor for the yield stress as a function of the strain rate according to Cowper–Symonds model (Equation (12)) [26] which parameters C = 396,500/s and *p* = 3.0745 were obtained from Boyce et al. (2007) [27]:

(12) $$\sigma =\sigma_{0}\left[1+{\left(\frac{\dot{\varepsilon }}{C}\right)}^{1/p}\right]$$

where *σ*_0_ is the quasi-static value of the yield stress, and *σ* is its generic dynamic value at strain rate $\dot{\varepsilon }$. Table 7 summerizes the parameters of the constitutive models.

#### 2.5.3. Validation of the Finite Element Discretization

**Figure 9:**
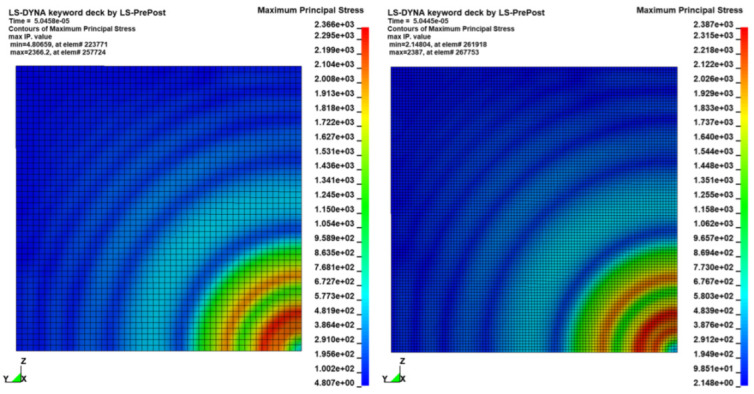
Stress field convergence. Comparison between the maximum principal stress field at 0.05 ms as predicted by the 2.5 mm model (**left**) and the 1.25 mm model (**right**). The difference in peak stress is less than 1%. No significant differences are visible. The stress wave is axially symmetric despite the squared shape of the arbitrary loaded surface.

**Figure 10:**
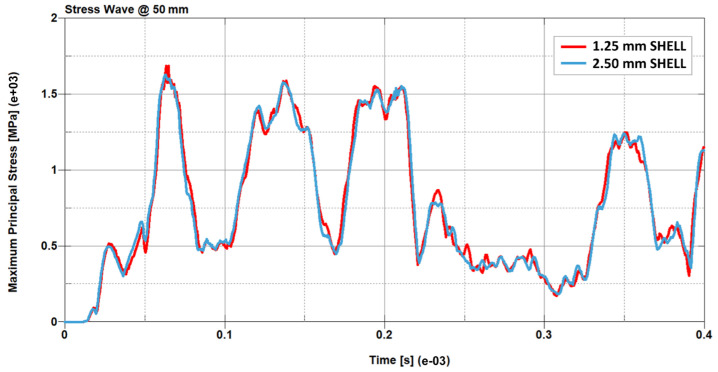
Maximum principal stress waves comparison at 50 mm from the origin. The predictions obtained by the higher nodal density model (red) and by the lower nodal density model (blue) are almost impossible to distinguish, even after 0.4 ms, which corresponds to around ten times the load application time. The maximum difference in peak stress is 10% after 0.23 ms, therefore 0.19 mm after the end of the loading time. This demonstrates that the stress prediction is consistent.

**Figure 11:**
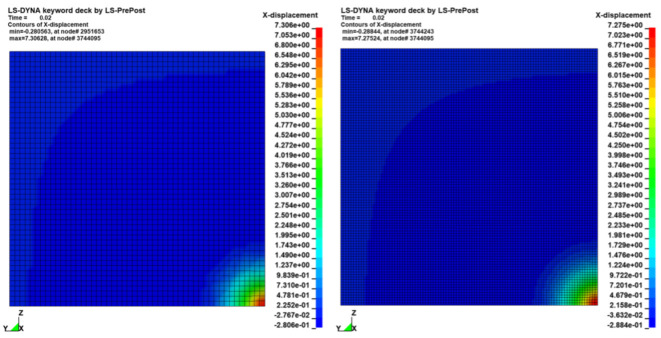
Residual deformation fields at 20 ms after the impacts [mm]. Comparison between the 2.5 mm shell model (**left**) and the 1.25 mm shell model (**right**). The predictions are equivalent; the maximum difference is less than 0.5% at the peak: 0.03 mm over 7.3 mm of maximum deformation.

To verify the consistency of the results in terms of stress waves and deformation fields, sensitivity tests were conducted on models with different mesh sizes. In this subsection we compare the results of a single Creusabro^®^ 8000 steel plate impacted by a monolithic .308 bullet at 735 m/s, simulated by applying the corresponding estimated load history to the sharpest distribution (100 mm^2,^ Figure 8). The results of the 2.5 mm shell model were compared with the results of an analogous model with 1.25 mm mesh size, therefore four times the nodal density. The comparisons show almost perfect adherence of the stress fields (Figure 9), stress waves (Figure 10), and residual displacement fields (Figure 11), validating the 2.5 mm shell discretization as an effective compromise between detailed load description, consistency of the results, and maximization of the time integration step for the containment of the computational cost in real-world industrial applications requiring the modelling of large structural systems.

## 3. Results

This section provides the experimental, analytical, and numerical results of the study. In Section 3.1 the pictures of the impacted plates and the frames capturing the evolution of the impacts are investigated to verify which of the considered impacts are compliant with the theoretical hypotheses of the bullet-splash. Section 3.2 summarizes the results of the measurements and estimations of the impact velocities. In Section 3.3 the load histories due to bullet-splash are plotted, having been calculated according to the theory from the measured impact velocities and the sections of the bullets. In Section 3.4, to verify the representativeness of the estimated load histories, the experimental fields of residual displacement are compared with the corresponding predictions obtained by means of explicit finite element simulations, where the discretized plates are loaded by direct application of the corresponding load curves.

### 3.1. Verification of the Bullet Splash Hypotheses

The verification of the hypotheses was performed by means of analyses of the experimental evidence. The inspection of the back plates allowed the verification of the non-penetration hypothesis. The inspection of the front plates allowed the verification of partial penetrations, here defined as the penetration of bullet material only through the front plate. The inspection of the full fragmentation of the bullets was performed by observation of the frames collected by means of the two high-frame-rate cameras in order to verify the gradual fragmentation of the bullet during the impact, the overall axial symmetry of the deflection kinematics, and the absence of major fragments rebounding from the plate and/or showing dimensions comparable with the bullets. A fragment was considered major if showing dimensions comparable with the calibre of the bullet. The presence of major fragments adhering to the impact surface was also verified by inspecting the plates.

#### 3.1.1. Non-Penetration

**Figure 12:**
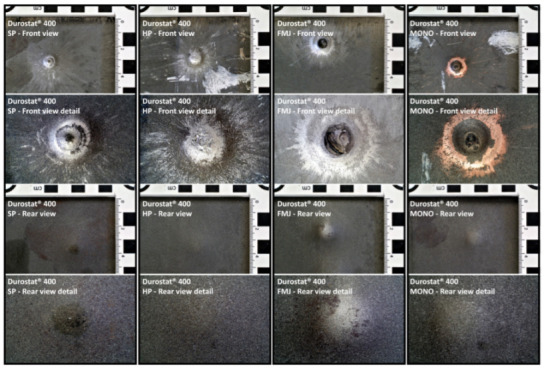
Front and rear surfaces of the Durostat^®^ 400 plates impacted by the bullets. The impacted areas show the marks of the interaction with the bullets, which can be distinguished in a central area where the interaction appears to be more compressive, and a peripheral area where the radial marks show the effects of the sliding of the bullet debris. The sliding marks are evident from a diameter of approximately 11 to 16 mm (roughly 100 mm^2^ to 200 mm^2^), to a diameter of 20 to 25 mm around the epicenter (around 400 mm^2^). The detailed front view of the FMJ effects shows the melted filler mixed with parts of the jacket, still blocked inside the crater.

**Figure 13:**
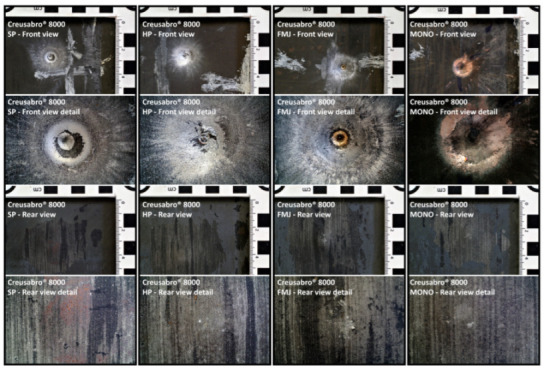
Front and rear surfaces of the Creusabro^®^ 8000 plates impacted by the bullets. The impacted areas show the marks of the interaction with the bullets, that can be distinguished in a central area where the interaction appears to be more compressive, and a peripheral area where the radial marks show the effects of the sliding of the bullet debris. The sliding marks are evident from a diameter of approximately 11 to 16 mm (roughly 100 mm^2^ to 200 mm^2^), to a diameter of 20 to 25 mm around the epicenter (around 400 mm^2^).

**Figure 14:**
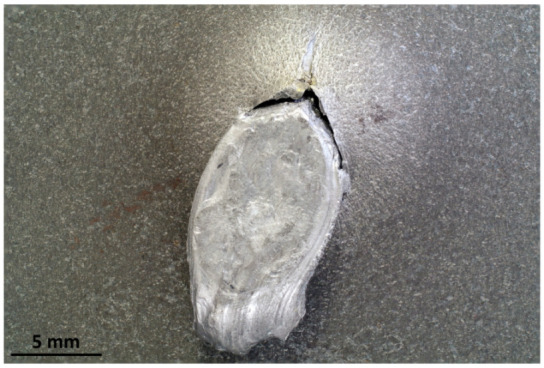
Partial penetration of FMJ bullet material through the first plate of Durostat^®^ 400. Back of the first plate showing the penetration of some melted filler material.

The targets were able to withstand the ballistic impacts without allowing any part of the bullets to penetrate through the 8 mm thickness. Figure 12 and Figure 13 show the effects of the impacts on the front and rear surfaces of the plates: the front views show the impact epicenter and the surrounding traces due to the radial debris deflection; the rear views show the bulges due to the residual plastic strain of the plates with no marks of penetration. It is worth noting that the Full Metal Jacket (FMJ) bullet caused the partial penetration of the first Durostat^®^ 400 plate (Figure 14).

#### 3.1.2. Full Fragmentation

**Figure 15:**
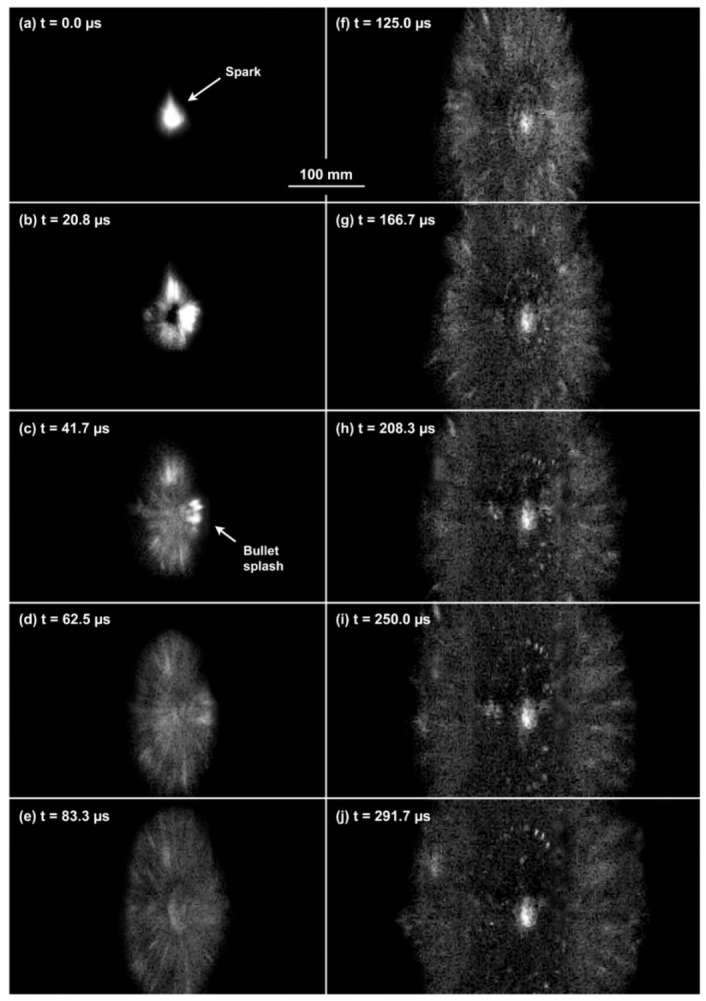
The figure shows ten frames of the first 0.3 ms after the impact for the combination FMJ-Creusabro^®^ 8000, taken from the footage recorded by the perspective camera (Camera 2). The first frames (**a**–**c**) show the crushing of the bullet at its first contact with the target, allowing the appreciation of a spark caused by the high hardness of the plate. Fragments are then projected radially from the impact point starting from small particles (**c**–**f**), with slightly bigger fragments later ricocheting in annular patterns (**f**–**j**). Frames shown here are processed to show the parts of interest, filtering out the noise otherwise preventing the appreciation of the bullet fragmentation.

**Figure 16:**
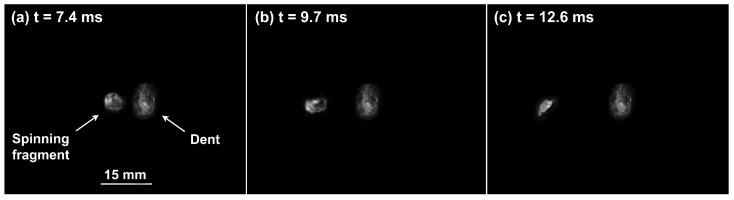
The figure shows a large fragment of the Full Metal Jacket bouncing back from the impact area of the Creusabro^®^ 8000 plate. The shot is therefore non-compliant with the bullet-splash definition. The fragment translates with perpendicular speed measured at 4.4 ± 2% m/s. Mild spinning can be appreciated. The three frames shown here (**a**–**c**), extracted from the second camera footage, were processed to isolate the fragment from the dust created after the impact: for each frame, only the fragment and the dent on the plate are visible; an approximate dimensional reference for the dent and the fragment is reported in frame (**a**). The fragment has a diameter of around 7.5 mm and thickness of about 4 mm, with the shape similar to the bulge created on the plate.

The analysis of the captured frames allowed to assess that all the bullets encountered full fragmentation and deflection, except for the FMJs. In fact, the FMJ impacting on Durostat^®^ 400 partially penetrated the first plate, while part of the FMJ impacting on Creusabro^®^ 8000 (Figure 15) was observed to slowly rebound after the impact, as a solid volume (Figure 16). These cases are therefore not compliant with the proposed bullet-splash definition.

### 3.2. Impact Velocity Estimation

The frame-by-frame analysis of the bullet kinematics before the impacts allowed identification of the impact velocity with a maximum error of ±5.7% (Table 8), except for shot 2 where the lack of light made the approach too uncertain. In this case the impact velocity was therefore estimated based on the muzzle velocity and the average percentage of speed loss calculated on four other Hollow Point bullets shot the same day in the same conditions (Table 9). The maximum error for shot 2 was therefore calculated as the sum of the maximum error due to muzzle velocity measurement (±2.8%) and the maximum error due to frame-by-frame approach on hollow point bullets (±5.7%). The maximum error associated with impact velocity of Shot 2 is therefore ±8.5%.

### 3.3. Estimated Load Histories

**Figure 17:**
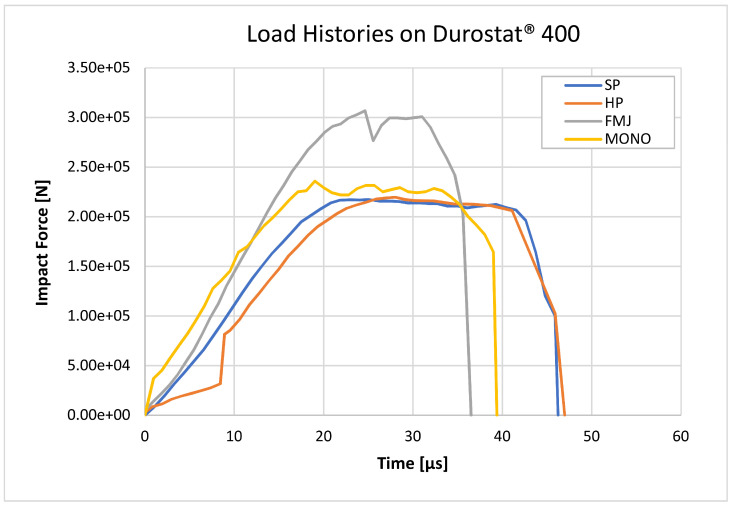
Load histories estimated according to Equation (8), applied to the impacts on Durostat^®^ 400. The SP, HP and MONO bullets have similar force peaks around 220–230 kN. The FMJ reaches instead 307 kN force. The duration of the impulses ranges from 36 to 47 µs.

**Figure 18:**
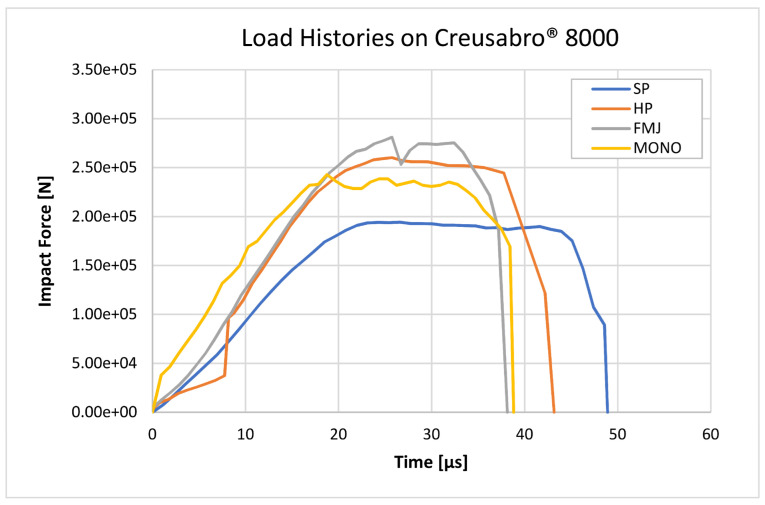
Load histories estimated according to Equation (8), applied to the impacts on Creusabro^®^ 8000. The SP reaches 194 kN peak force, MONO reaches 243 kN, HP reaches 260 kN, and FMJ reaches 281 kN. The durations of the impulses range from 39 µs for FMJ to 49 µs for SP.

According to the proposed load history approach, eight load histories (Figure 17 and Figure 18) were calculated, based on the bullets’ section (Figure 1) and the measured impact velocities (Table 8). The mass distribution was reconstructed based on the scaled sections associated with the density values summarized in Table 10, so that the volume integration of the mapped density fields exactly equals the nominal masses of the bullets, and therefore the time integration of the load histories correctly corresponds to the momentum of the impacting bullets.

### 3.4. Field of Residual Displacements

**Figure 19:**
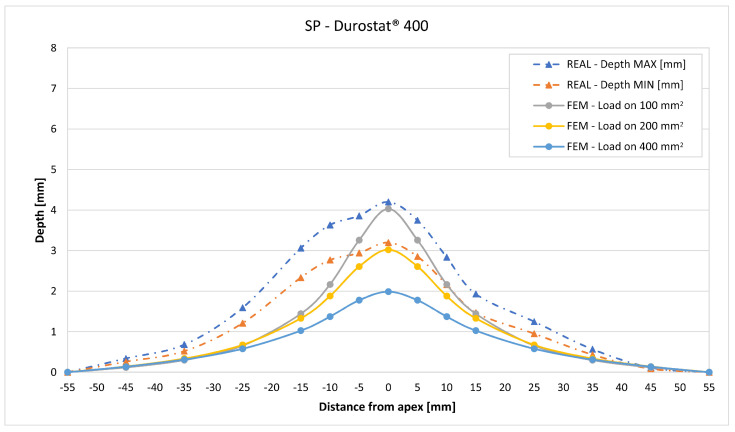
Residual displacements caused by the Soft Point bullet on Durostat^®^ 400 plates. The predicted apex displacement for the 100 mm^2^ distribution (grey line) is near the maximum experimental value (blue dotted line), around 4 mm. The predicted apex displacement for the 200 mm^2^ distribution (yellow line) is near the minimum experimental value, around 3 mm. The predicted apex displacement for the 400 mm^2^ distribution (light blue line) is around 1 mm lower than the minimum experimental value. The radial extension of the bulge is underestimated. Over 35 mm radial distance from the apex the three load distributions are impossible to distinguish.

**Figure 20:**
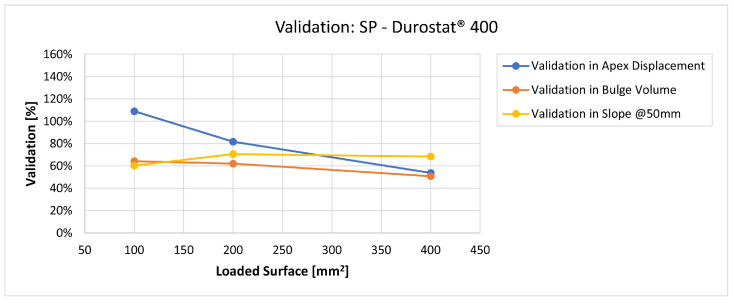
Soft Point bullet on Durostat^®^ 400 plates: validation of the calculated residual displacement fields in terms of apex displacement (blue), total bulge volume (red), and slope at 50 mm (yellow). In abscissa the loaded area of the three arbitrary distributions. In terms of apex displacement, the 100 mm^2^ distribution gives the best estimation (109%), while the 200 mm^2^ and 400 mm^2^ give 82% and 55%. The three distributions provide more consistent predictions in terms of bulge volume and slope, all between 51 and 68%.

**Figure 21:**
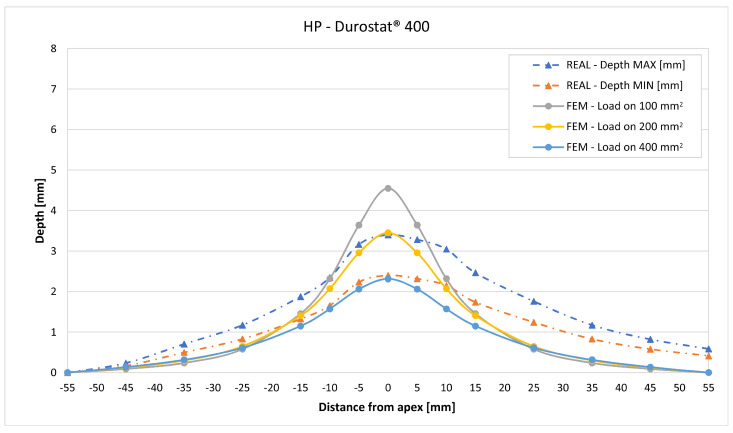
Residual displacements caused by the Hollow Point bullet on Durostat^®^ 400 plates. The predicted apex displacement for the 100 mm^2^ distribution (grey line) exceeds the maximum experimental value (blue dotted line) of about 1 mm. The predicted apex displacement for the 200 mm^2^ distribution (yellow line) coincides with the maximum experimental value, around 3.4 mm. The predicted apex displacement for the 400 mm^2^ distribution (light blue line) is almost coincident with the minimum experimental value. The radial extension of the bulge is slightly underestimated. Over 25 mm radial distance from the apex the three load distributions are almost impossible to distinguish.

**Figure 22:**
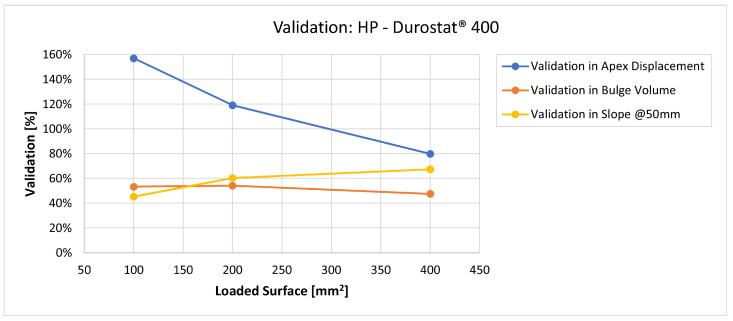
Validation for HP on Durostat^®^ 400: validation of the calculated residual displacement fields in terms of apex displacement (blue), total bulge volume (red), and slope at 50 mm (yellow). In abscissa the loaded area of the three arbitrary distributions. In terms of apex displacement, the 200 mm^2^ distribution gives the best estimation (119%), while the 100 mm^2^ and 400 mm^2^ give 157% and 80%. The three distributions provide more consistent predictions in terms of bulge volume and slope, all between 45% and 67%.

**Figure 23:**
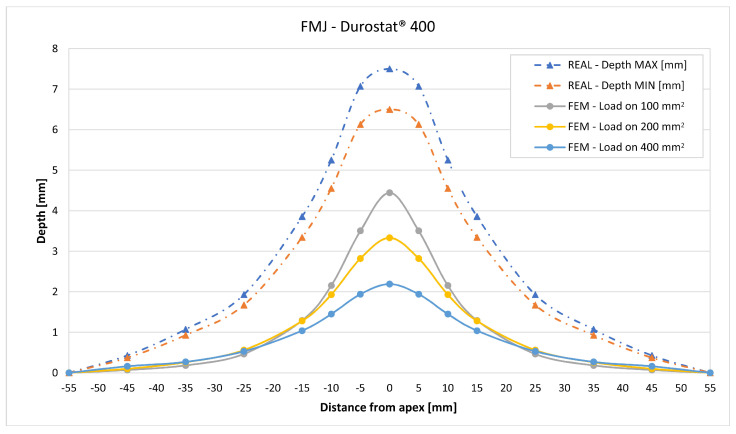
Residual displacements caused by the Full Metal Jacket bullet on Durostat^®^ 400 plates. The predicted apex displacement for the 100 mm^2^ distribution (grey line) underestimates the experimental range (blue and red dotted lines) of about 2.5 mm. The overall bulge extension is significantly underestimated. For radial distance over 25 mm from the apex, the three load distributions are almost impossible to distinguish.

**Figure 24:**
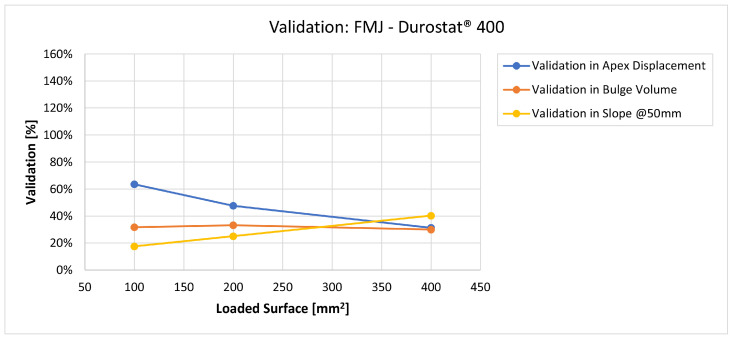
Validation for FMJ on Durostat^®^ 400: validation of the calculated residual displacement fields in terms of apex displacement (blue), total bulge volume (red), and slope at 50 mm (yellow). In abscissa the loaded area of the three arbitrary distributions. In terms of apex displacement, the 100 mm^2^ distribution gives the best estimation (63%), while the 200 mm^2^ and 400 mm^2^ give 48% and 30%. The three distributions provide consistent predictions in terms of bulge volume around 30–33%. In slope, the estimate increases as the distribution area, from 17.5% to 40%.

**Figure 25:**
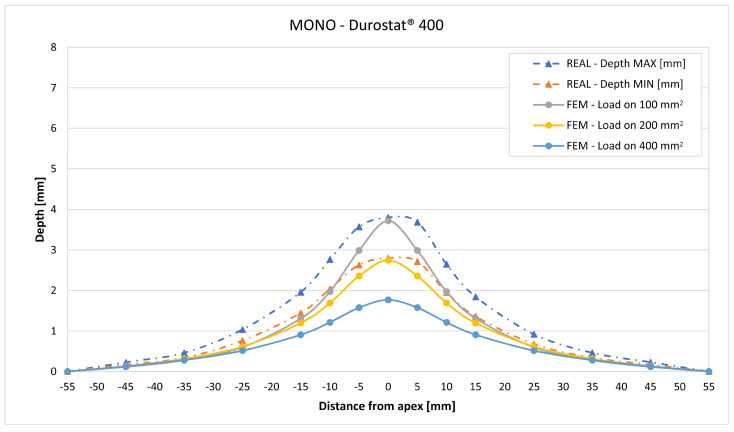
Residual displacements caused by the Monolithic bullet on Durostat^®^ 400 plates. The predicted apex displacement for the 100 mm^2^ distribution (grey line) is almost coincident with the maximum experimental value (blue dotted line). The predicted apex displacement for the 200 mm^2^ distribution (yellow line) coincides with the minimum experimental value. The radial extension of the bulge is slightly underestimated. For radial distances over 35 mm from the apex, the three load distributions are impossible to distinguish.

**Figure 26:**
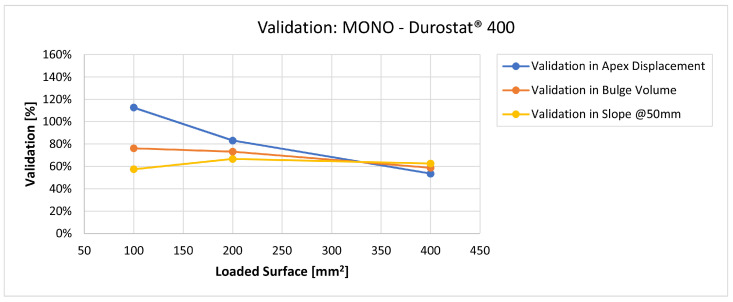
Validation for MONO on Durostat^®^ 400: validation of the calculated residual displacement fields in terms of apex displacement (blue), total bulge volume (red), and slope at 50 mm (yellow). In abscissa the loaded area of the three arbitrary distributions. In terms of apex displacement, the 100 mm^2^ distribution gives the best estimation (113%), while the 200 mm^2^ and 400 mm^2^ give 83% and 54%. The bulge volume predictions decrease from 76% to 63% as the area increases. The slope predictions are more consistent, between 57% and 67%.

**Figure 27:**
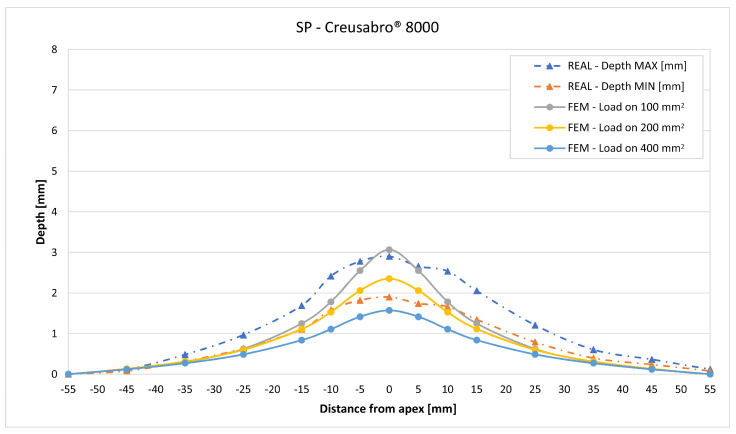
Residual displacements caused by the Soft Point bullet on Creusabro^®^ 8000 plates. The predicted apex displacement for the 100 mm^2^ distribution (grey line) slightly exceeds the maximum experimental value (blue dotted line). The predicted apex displacement for the 200 mm^2^ distribution (yellow line) is in the middle of the experimental range. The predicted apex displacement for the 400 mm^2^ distribution (light blue line) underestimates the minimum experimental value of about 0.5 mm. The radial extension of the bulge is slightly underestimated. Over 35 mm radial distance from the apex the three load distributions are impossible to distinguish.

**Figure 28:**
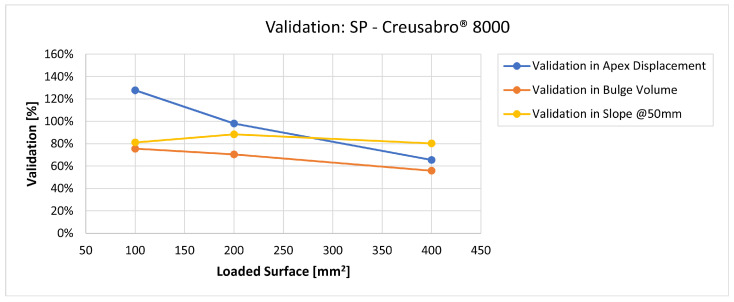
Validation for SP on Creusabro^®^ 8000. Validation of the calculated residual displacement fields in terms of apex displacement (blue), total bulge volume (red), and slope at 50 mm (yellow). In abscissa the loaded area of the three arbitrary distributions. In terms of apex displacement, the 200 mm^2^ distribution gives the best estimation (98%), while the 100 mm^2^ and 400 mm^2^ give 128% and 66%. The bulge volume predictions decrease from 81% to 56% as the area increases. The slope predictions are more consistent, between 80% and 88%.

**Figure 29:**
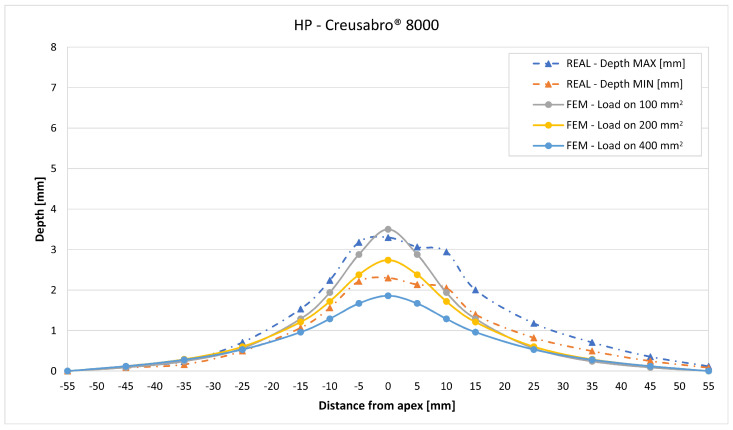
Residual displacements caused by the Hollow Point bullet on Creusabro^®^ 8000 plates. The predicted apex displacement for the 100 mm^2^ distribution (grey line) exceeds the maximum experimental value (blue dotted line) of about 0.2 mm. The predicted apex displacement for the 200 mm^2^ distribution (yellow line) is in the middle of the experimental range. The predicted apex displacement for the 400 mm^2^ distribution (light blue line) is 0.5 lower than the minimum experimental value. The radial extension of the bulge is slightly underestimated. For radial distances over 25 mm from the apex, the three load distributions are impossible to distinguish.

**Figure 30:**
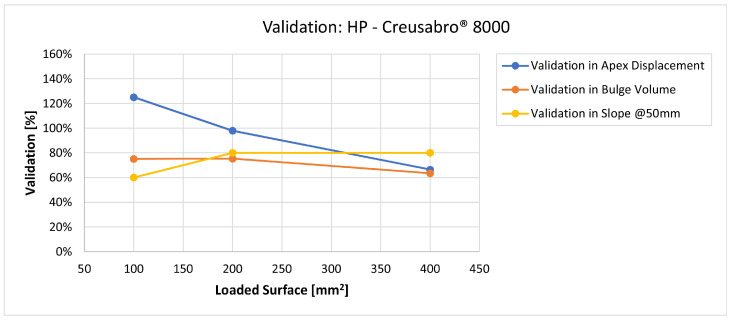
Validation for HP on Creusabro^®^ 8000. Validation of the calculated residual displacement fields in terms of apex displacement (blue), total bulge volume (red), and slope at 50 mm (yellow). In abscissa the loaded area of the three arbitrary distributions. In terms of apex displacement, the 200 mm^2^ distribution gives the best estimation (98%), while the 100 mm^2^ and 400 mm^2^ give 125% and 63%. The bulge volume predictions decrease from 75% to 63% as the area increases. The slope predictions increase from 60% (100 mm^2^) to 80% (200 and 400 mm^2^).

**Figure 31:**
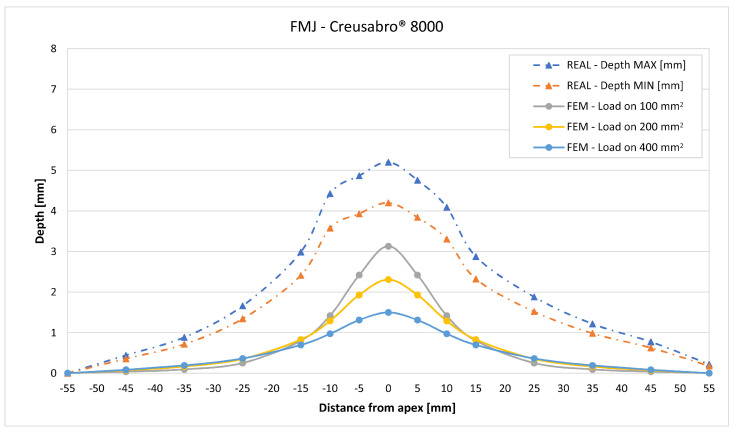
Residual displacements caused by the Full Metal Jacket bullet on Creusabro^®^ 8000 plates. The predicted apex displacement for the 100 mm^2^ distribution (grey line) underestimates the experimental (blue and red dotted lines) range of about 2 mm. The overall bulge extension is significantly underestimated. For radial distances over 15 mm from the apex, the three load distributions are difficult to distinguish.

**Figure 32:**
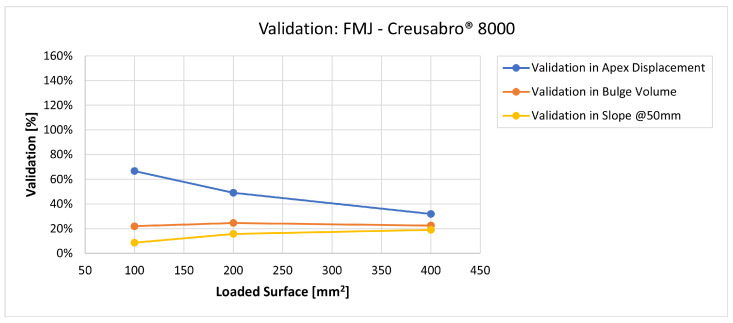
Validation for FMJ on Creusabro^®^ 8000. Validation of the calculated residual displacements fields in terms of apex displacement (blue), total bulge volume (red), and slope at 50 mm (yellow). In abscissa the loaded area of the three arbitrary distributions. In terms of apex displacement, the 100 mm^2^ distribution gives the best estimation (67%), while the 200 mm^2^ and 400 mm^2^ give 49% and 32%. The bulge volume predictions are consistent between 22% and 25%. The slope predictions increase from 9% (100 mm^2^) to 19% (400 mm^2^).

**Figure 33:**
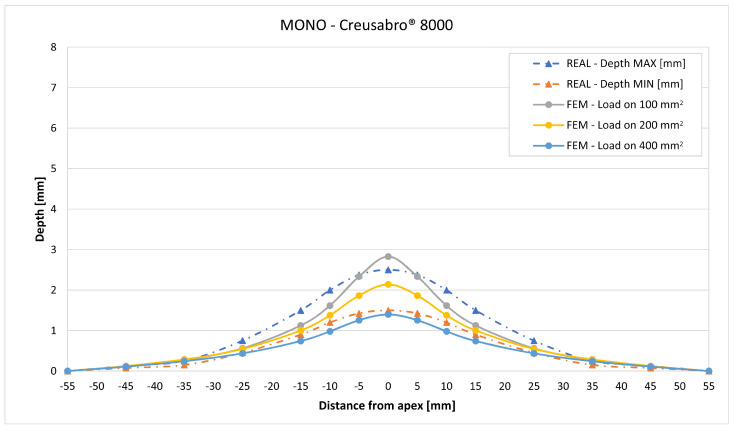
Residual displacements caused by the Monolithic bullet on Creusabro^®^ 8000 plates. The predicted apex displacement for the 100 mm^2^ distribution (grey line) exceeds the maximum experimental value (blue dotted line) by 0.3 mm. The predicted apex displacement for the 200 mm^2^ distribution (yellow line) almost coincides with the middle range of the experimental values (blue and red dotted lines). The predicted apex displacement for the 400 mm^2^ distribution (light blue line) coincides with the minimum experimental value. The radial extension of the bulge is well estimated. For radial distances over 35 mm from the apex, the three load distributions are impossible to distinguish.

**Figure 34:**
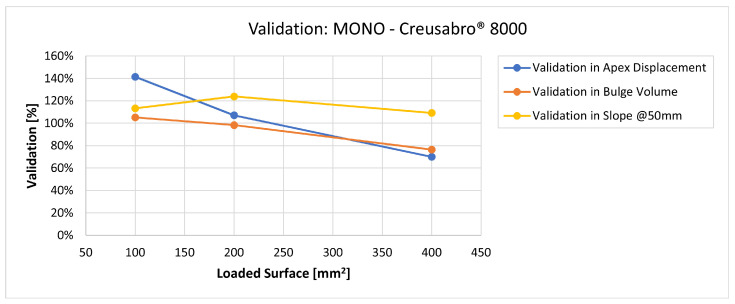
Validation for MONO on Creusabro^®^ 8000. Validation of the calculated residual displacements fields in terms of apex displacement (blue), total bulge volume (red), and slope at 50 mm (yellow). In abscissa the loaded area of the three arbitrary distributions. In terms of apex displacement, the 200 mm^2^ distribution gives the best estimation (107%), while the 100 mm^2^ and 400 mm^2^ give 141% and 70%. The bulge volume predictions decrease with the area, from 113% to 76%. The slope predictions are more consistent, between 109% and 123%.

In Figure 19, Figure 20, Figure 21, Figure 22, Figure 23, Figure 24, Figure 25, Figure 26, Figure 27, Figure 28, Figure 29, Figure 30, Figure 31, Figure 32, Figure 33 and Figure 34 we provide the comparison between numerical and experimental results in terms of residual displacements of the plates. The fields of residual displacements have been represented as a function of the radial distance from the apex of the bulges (Figure 19, Figure 21, Figure 23, Figure 25, Figure 27, Figure 29, Figure 31 and Figure 33). The experimental measurements (represented with their variability due to a ±0.5 mm error in positioning the instrument at the bulge apex) are compared with the simulation results obtained with the three arbitrary load distributions, where the same load history was distributed on 100, 200, and 400 mm^2^ as described in Section 2.5.1. Based on the mapping of the residual displacements, three validation indexes have been considered: the apex displacement, the overall volume of the residual bulge, and the radial slope at 50 mm from the apex (the boundary of the measured range). The validation was conducted by dividing the indexes calculated on the simulation results by the indexes calculated on the experimental results. The validation results are presented as percentages, so that a validation higher than 100% means that the simulation overestimated the corresponding experimental index. To enhance the effects of the load distribution on the validation indexes, the three indexes were plotted as a function of the loaded area (Figure 20, Figure 22, Figure 24, Figure 26, Figure 28, Figure 30, Figure 32 and Figure 34).

## 4. Discussion

All the observed shots showed massive fragmentation of the bullets, with no full penetration of the 8 mm thick targets. However, evidence of non-complete fragmentation was reported on both FMJ bullets: a large solid part of the bullet, with dimensions comparable with the caliber, was found bouncing back after the shot on Creusabro^®^ 8000 (Figure 16), and a significant volume of filler material together with part of the jacket material was found having penetrated the first plate of Durostat^®^ 400 (Figure 12 and Figure 14). These two cases, therefore, do not satisfy the theoretical hypotheses supporting the formula for the load history estimation demonstrated in Section 2.4, For this reason both FMJ shots should be considered control cases to evaluate the effectiveness of the method when its applicability conditions are not verified.

Considering the fields of residual displacement, the simulation and the experimental results diverge significantly for both FMJ bullet cases. The predicted displacement fields are being consistently and significantly underestimated, particularly so in terms of bulge volume and slope, for which the best estimations for FMJ impacts are, respectively, 33% and 40% on Durostat^®^ 400 and 25% and 19% for Creusabro^®^ 8000 (Figure 23, Figure 24, Figure 31 and Figure 32). The relevant underestimation in the fields of residual displacement in these two cases is due to the force peaks needed to decelerate the relevant solid parts of the bullets instead of gradually deflecting their mass as happens during the ideal bullet splash. In the case of the partial penetration of the Durostat^®^ 400 plate, the local reduction in the stiffness of the target due to the loss of integrity of the front plate and the direct impact of parts of the bullet onto the back plate should also have an effect in increasing the real back plate deformation.

The experimental verifications have instead confirmed that, on the contrary, the SP, HP, and MONO cases are fully compliant with the bullet-splash theoretical hypotheses; the comparisons between the predicted and experimental residual displacement fields for these cases are therefore considered significant for the validation of the load histories estimated with the proposed method.

The validation was performed on the results obtained by simulating the impacts on the three different arbitrary load distributions considered representative of the extension of the marks left on the plates after the impacts. The 100 mm^2^ and 200 mm^2^ distributions were considered representative of the minimum and maximum observed areas where the interaction between bullet and target is mainly compressive, causing the formation of the fragments and their 90-degree, almost instantaneous, deflection. The 400 mm^2^ distribution area, instead, includes approximately the entire extension of the interaction areas observed on the plates, including the peripheral area where the radial marks show that the interaction between bullet and target only consisted in a sliding contact of the already formed and deflected fragments. This last distribution was therefore expected to be less representative of the local compressive phenomena, but still relevant as a sensitivity test case of the effects of the pressure distributions on the deformation fields.

The comparisons between the simulated and experimental fields of residual displacements (Figure 19, Figure 21, Figure 23, Figure 25, Figure 27, Figure 29, Figure 31 and Figure 33) show that the effects of the load distributions are significant only within 25 to 35 mm from the apex. The differences in terms of residual displacement at the apex are due to the fact that the three distributions apply pressure intensities that are inversely proportional to their loaded areas to guarantee that the integration of the pressure fields is equivalent to the load history for all the arbitrary distributions. To apply the same force, the pressure applied on the 100 mm^2^ area must be, respectively, two and four times higher than the intensity applied by the 200 mm^2^ and 400 mm^2^ uniform distributions; therefore, the loaded elements must withstand proportionally higher stress and consequently encounter higher fields of plastic strain compared to the same elements loaded with lower intensity distributions. This explains the local differences in terms of maximum residual displacement at the apex between the three force-equivalent distributions as well as the almost indistinguishable effects over 25 to 35 mm from the apex, since the peripheral elements encounter approximately the same stress waves for all the distributions. In fact, the differences in radial extension between the three loaded areas are very limited compared to the distance covered by a mechanical signal during the duration of the considered impulses, therefore the stress waves propagating from the 100 mm^2^ distribution have a duration that is less than 3% shorter than the 400 mm^2^ one, which has neglectable engineering effects.

The validation graphs (Figure 20, Figure 22, Figure 24, Figure 26, Figure 28, Figure 30, Figure 32 and Figure 34) demonstrate that the estimated load histories applied on the 100 mm^2^ and 200 mm^2^ distributions allow correct predictions of the residual displacements at the apex for all the verified bullet splashes (SP, HP, and MONO), with the sharpest distribution giving percentage validations between 109% and 157% (average 126%) on Durostat^®^ 400 and between 125% and 141% (average 131%) for Creusabro^®^ 8000. Moreover, the 200 mm^2^ distribution gives representative validations between 82% and 119% (average 95%) on Durostat^®^ 400 and between 98% and 107% (average 101%) for Creusabro^®^ 8000. It is crucial to notice that even though the two sharpest distributions have a 100% difference in pressure intensity and loaded surfaces, the average difference in the estimations of the maximum residual displacement between the two is limited to 31% (129% vs. 98%). Even the 400 mm^2^ distribution gives an average validation in apex displacement of 65%, despite a pressure field intensity equal to 25% of the one imposed by the sharpest distribution.

In all of the cases, the validation in bulge volume is much more consistent between the distributions, with just a modest reduction from the sharpest to the flattest. The sharpest distribution gives average percentage validations of 65% on Durostat^®^ 400 and 85% on Creusabro^®^ 8000, while the 200 mm^2^ distribution gives 63% on Durostat^®^ 400 and 81% on Creusabro^®^ 8000; the flattest gives 52% on Durostat^®^ 400 and 65% on Creusabro^®^ 8000. The slight reduction in bulge volume is due to the reduction in local stress that causes the reduction in the maximum residual displacement at the apex, which has a minor effect on the total bulge volume.

The validation in slope at 50 mm from the apex in most of the cases increases from the sharpest to the intermediate distribution and then slightly decreases or remains constant for the flattest: the 100 mm^2^ gives an average validation of 54% on Durostat^®^ 400 and 85% on Creusabro^®^ 8000, while the 200 mm^2^ distribution gives 66% on Durostat^®^ 400 and 97% on Creusabro^®^ 8000. The 400 mm^2^ gives 66% on Durostat^®^ 400 and 90% on Creusabro^®^ 8000. The increase and slight decrease with the extension of the loaded areas is explained by the opposite effects of reducing the load intensity, which decreases the bulge volume, and reducing the average distance from the load application to the peripheral area where the slope is calculated, which tends to increase the peripheral deformation.

In terms of general bulge shape, the simulations tend to underestimate the half-depth diameter. The calculation of the bulge volumes shows that the simulations of the shots on Durostat^®^ 400 estimate bulge volumes around 35% lower than the reality (Table 11), while on Creusabro^®^ 8000 the estimation is better, with just 15% of underestimation on average between the cases (Table 12). A difference in shape between the plate materials is also observed in terms of slope of the bulge at 50 mm from the apex, with a 46% underestimation on Durostat^®^ 400 plates (Table 11) and a 15% underestimation on Creusabro^®^ 8000 (Table 12), again on average between the cases. These differences are due to the simplification hypotheses introduced with the method, in particular with the hypothesis of a 90-degree deflection of the bullet debris, which is an ideal reference case that is closer to reality when the impact surface keeps its perfect planarity. In reality, after the impact surface gradually bulges, the debris are subjected to a slightly higher deflection angle, interacting on a gradually wider surface on the target. This results in a radial component of the impact force that increases the width of the bulge and a slight increase in the normal force due to the higher angle of deflection of the debris. This is confirmed by the fact that the estimation of the bulge volume and slope is significantly better on Creusabro^®^ 8000 plates, which encounter more contained residual displacements compared to the Durostat^®^ 400 ones, thanks to its higher hardness and tensile strength. This confirms that the proposed method is well suited for targets much harder than the bullets, which represents the interactions between bullets and a perfectly working ballistic protection.

## 5. Conclusions

The study verified the effectiveness of the proposed simplified formula to estimate the load history due to bullet-splash in predicting the resultant interaction forces due to semi-jacketed, full metal jacket and monolithic .308 Winchester bullets impacting on double 4 mm-thick plates of Durostat^®^ 400 and Creusabro^®^ 8000 hard steels.

The experimental analyses reported that the SP, HP and MONO impacts were compliant with the bullet-splash theory, while both the FMJ cases failed to meet the theoretical hypotheses due to partial penetration of the target and partial fragmentation. None of the targets were fully penetrated.

The validation of the method was performed by comparing the experimental results, in terms of fields of residual displacement measured on the impacted plates, with the results of finite element explicit simulations to predict the residual deformations of the plates subjected to the load histories estimated according to the proposed theory. Sensitivity tests were conducted to verify the influence of the extension of the load distribution on the numerical results.

The validation in terms of residual displacements at the apex confirm the effectiveness of the method for all the shots fully compliant with the bullet-splash hypotheses (SP, HP, MONO). On the contrary, the simulations applying the load histories estimated for the FMJ bullets significantly underestimated the residual deformation fields, confirming that the effectiveness of method strictly depends on the validation of its theoretical hypotheses.

The validation in terms of bulge volume and slope at 50 mm from the apex of the bulges shows an underestimation of the overall residual deformation fields which has been related to the strength of the target: the more the target is deformed under the normal impact forces, the more the debris must be deflected, therefore probably resulting in a wider bulge than expected by considering only a 90-degree deflection as the method hypothesizes. This suggests a possible improvement and generalization of the formula to take into account deflection angles higher than 90 degrees, depending on the specific experimental evidence.

The sensitivity tests conducted to check the effects of the load distributions on the deformation fields demonstrate that even varying the area (or intensity) by two or four times only causes local effects, within 25 to 35 mm from the apex, while the bulge volume and the slope at 50 mm are just slightly influenced. It is worth noting that the results confirm that the two sharpest distributions (100 mm^2^ and 200 mm^2^) are the most representative, with validations in terms of apex displacements between 82% and 157%. These two distributions were identified as the range of areas subjected to mostly compressive interactions according to the marks left on the plates; this suggests that an equivalent application area could be effectively identified in this range for all the test cases. The consistency of the validation percentages for different bullets with significantly different mechanical and geometrical characteristics further confirms that the real space distribution of the pressure fields has neglectable effects, and therefore the resultant load history approach is appropriate for the purpose of the study.

All the evidence suggests that the method could be properly applied to the assessment of structural systems subjected to ballistic impacts, provided that previous experimental ballistic tests have been analyzed and demonstrated that the target surface is able to completely fragment and deflect the impactor without allowing any penetration. In case of significant deformation of the impact surface, the results suggest that the method should be applied more carefully, with conservative safety margins to be assessed considering the specific outcomes of the experimental tests.

Further developments of the research should therefore investigate the effects of high deflection angles and the influence of the debris dimensions on the intensity of the impulses, with the ultimate goal of identifying possible simplified procedures to generalize the load history approach and to allow the parsimonious simulation of a wider range of ballistic impacts causing partial penetrations and fragmentations or high deformation of the target.

## Figures and Tables

**Table 1:** Main data of the bullets used for experimentation as provided by the manufacturers. The nominal muzzle velocity is the expected velocity at the exit of the firearm. The nominal energy is the expected kinetic energy of the bullets at the exit of the firearm.

Bullet Type	Cal.	Bullet Mass [g]	Nominal Muzzle Velocity [m/s]	Nominal Energy [J]
Soft Point (SP)	308 Win	11.7	870	4428
Hollow Point (HP)	308 Win	10.9	800	3485
Full Metal Jacket (FMJ)	308 Win	9.5	865	3570
Monolithic (MONO)	308 Win	9.6	860	3550

**Table 2:** Chemical analyses of Creusabro^®^ 8000 steel (wt%).

	C	S	P	Si	Mn	Ni	Cr	Mo
Metal Plate	0.21	0.002	0.009	0.74	1.18	0.49	0.70	0.26
Steel Mill Specs	0.28 Max	0.005 Max	0.018 Max	Unspecified	1.60 Max	1.00 Max	1.60 Max	0.40 Max

**Table 3:** Chemical analyses Durostat^®^ 400 steel (wt%).

	C	S	P	Si	Mn	Al	Cr	Mo	B	Ti
Metal Plate	0.10	0.002	0.008	0.16	2.08	0.03	0.26	<0.01	0.001	0.021
Steel Mill Specs	0.18 Max	0.010 Max	0.025 Max	0.60 Max	2.10 Max	0.02 Min	1.00 Max	0.50 Max	0.005 Max	0.050 Max

**Table 4:** Summary of tensile tests results for the Creusabro^®^ 8000 steel and Durostat^®^ 400 plates used for experimentation.

	E [Gpa]	YS [Mpa]	UTS [Mpa]	YS/UTS	Ag%	A%
Creusabro^®^ 8000	210	1190	1735	0.68	5	11
Durostat^®^ 400	210	1100	1290	0.85	3	11

**Table 5:** Data acquisition: camera 1.

Shot	Resolution [px]	Sampling Frequency [fps]
1	256 × 256	39,000
2	320 × 128	50,000
3	320 × 128	50,000
4	320 × 128	50,000
5	320 × 128	50,000
6	320 × 128	50,000
7	320 × 128	40,000
8	320 × 128	40,000

**Table 6:** Data acquisition: camera 2.

Shot	Resolution [px]	Sampling Frequency [fps]
1	320 × 256	33,000
2	320 × 152	48,000
3	320 × 152	48,000
4	320 × 152	48,000
5	320 × 152	48,000
6	320 × 152	48,000
7	320 × 152	48,000
8	320 × 152	48,000

**Table 7:** Table of the constitutive parameters associated with the Creusabro^®^ 8000 and Durostat^®^ 400 models associated with the plates: RO = density; E = Young Modulus; PR = Poisson Ratio; SIGY = Yield Stress; ETAN = linear hardening modulus; EPPF = initial necking strain; C, p = rate effect parameters according to the Cowper–Symonds model; EPPFR = ultimate failure strain.

	RO [kg/m^3^]	E [GPa]	PR	SIGY [MPa]	ETAN [MPa]	EPPF	C [s^−1^]	p	EPPFR
Creusabro^®^ 8000	7800	210	0.33	1190	11,891.7	0.058	396,500	3.0745	0.11
Durostat^®^ 400	7800	210	0.33	1100	5759.6	0.044	396,500	3.0745	0.11

**Table 8:** Estimated impact velocities.

Shot	Bullet	Material	Impact Speed [m/s]	Maximum Error [%]
1	Soft Point (SP)	Durostat^®^ 400	642	1.1
2	Hollow Point (HP)	Durostat^®^ 400	645	8.5 *
3	Full Metal Jacket (FMJ)	Durostat^®^ 400	769	1.2
4	Monolithic (MONO)	Durostat^®^ 400	738	3.5
5	Soft Point (SP)	Creusabro^®^ 8000	607	1.2
6	Hollow Point (HP)	Creusabro^®^ 8000	702	5.7
7	Full Metal Jacket (FMJ)	Creusabro^®^ 8000	736	2.9
8	Monolithic (MONO)	Creusabro^®^ 8000	749	2.8

* Estimated based on the average velocity loss of four Hollow Point bullets shot in the same conditions.

**Table 9:** Average velocities at muzzle and at 100 m and relative losses for each bullet type. Averages are computed over a total of four shots per bullet type.

Bullet	$v_{muzzle}$ [m/s]	$v_{100m}$ [m/s]	Loss [%]
Soft Point (SP)	763	620	18.7
Hollow Point (HP)	814	727	10.7
Full Metal Jacket (FMJ)	843	731	13.3
Monolithic (MONO)	837	741	11.5

**Table 10:** Density values associated with the bullet materials for the four bullet types so that the volume integration of the density fields correspond to the nominal bullets’ masses.

Bullet	$\rho_{jacket}$ [kg/m^3^]	$\rho_{filler}$ [kg/m^3^]	Bullet Mass [g]
Soft Point (SP)	8730	12,640	11.66
Hollow Point (HP)	8730	12,280	10.89
Full Metal Jacket (FMJ)	8730	11,325	9.53
Monolithic (MONO)	9425	-	9.59

**Table 11:** Validation comparisons for Durostat^®^ 400. Validation percentages are calculated as numerical simulation results obtained for the 100 mm^2^ distribution divided by the experimental results.

	SP	HP	FMJ *	MONO
Validation in Bulge Volume	64.22%	53.31%	31.69%	76.12%
Validation in Apex Displacement	108.90%	156.92%	63.47%	112.62%
Experimental Slope @ 50 mm	−2.00%	−2.00%	−4.00%	−2.00%
Numerical Slope @ 50 mm	−1.20%	−0.91%	−0.70%	−1.15%
Validation in Slope @ 50 mm	60.19%	45.26%	17.50%	57.43%

* Not compliant with bullet-splash hypotheses.

**Table 12:** Validation comparisons for Creusabro^®^ 8000. Validation percentages are calculated as numerical simulation results obtained for the 100 mm^2^ distribution divided by the experimental results.

	SP	HP	FMJ *	MONO
Validation in Bulge Volume	75.48%	74.98%	21.95%	105.05%
Validation in Apex Displacement	127.73%	125.00%	66.62%	141.36%
Experimental Slope @ 50 mm	−1.50%	−1.50%	−4.50%	−1.00%
Numerical Slope @ 50 mm	−1.22%	−0.90%	−0.39%	−1.13%
Validation in Slope @ 50 mm	81.11%	60.00%	8.69%	113.26%

* Not compliant with bullet-splash hypotheses.

## Data Availability

The data presented in this study are available on request from the corresponding author.
